# Associations of cholinergic white matter hyperintensity volume with cognitive decline and incident dementia in older adults: a cohort study

**DOI:** 10.1186/s12877-025-06447-x

**Published:** 2025-10-10

**Authors:** Heyang Lu, Rui Li, Jialin Li, Kelin Xu, Yanfeng Jiang, Yingzhe Wang, Xingdong Chen, Mei Cui

**Affiliations:** 1 Department of Neurology, Huashan Hospital, Fudan University, No.12 Middle Wulumuqi Road, Shanghai, 200040 China; 2 Human Phenome Institute, Research and Innovation Center, Shanghai Pudong Hospital, Zhangjiang Fudan International Innovation Center, Fudan University, Zhangheng Road 666, Shanghai, 200120 China; 3 Department of Biostatistics, School of Public Health, Key Laboratory of Public Health Safety of Ministry of Education, Fudan University, Shanghai, China; 4 Fudan University Taizhou Institute of Health Sciences, Taizhou, Jiangsu China; 5 Department of Neurology and National Center for Neurological Disorders, Huashan Hospital, Fudan University, Shanghai, China; 6 State Key Laboratory of Brain Function and Disorders and MOE Frontiers Center for Brain Science, Shanghai Medical College, Fudan University, Shanghai, China

**Keywords:** Cholinergic degeneration, White matter hyperintensity, Cognitive dysfunction, Neuroimaging

## Abstract

**Background:**

Few studies take both the volume and location of white matter hyperintensities (WMHs) into account to explore the association between WMH burden within the cholinergic pathways and cognitive impairment. We aimed to investigate associations of cholinergic WMH volume (WMHV) with global cognitive function, cognitive decline, and incident dementia in older adults, which may help us identify a potential imaging biomarker.

**Methods:**

We assessed non-demented participants (*n* = 751, mean age 60 years) from the Taizhou Imaging Study with brain MRI at baseline and repeated measures of cognition over 5 years of follow-up. WMHV in the whole brain, the cholinergic pathways, and different tracts in the Montreal Neurologic Institute (MNI) standard space were analyzed. Linear regression, Cox regression, and partial correlation tests were performed to investigate associations between global and regional WMHV and cognitive outcomes.

**Results:**

During follow-up, cholinergic WMHV was associated with an annual decline of Mini-Mental State Examination (MMSE) (β coefficient, -0.239; *P* = 0.004), and incident dementia (HR = 3.54; 95%CI: 2.05–6.10). Within the cholinergic pathways, WMHs in corpus callosum and corona radiata were significantly related to incident dementia. Global WMHV was also associated with global cognitive decline (β coefficient, -0.049; *P* = 0.002). However, greater global WMHV only slightly increased the risk of incident dementia (HR = 1.23; 95%CI: 1.11–1.35). Neither global nor cholinergic WMHV was associated with MMSE in cross-sectional analysis.

**Conclusions:**

Cholinergic WMHV is associated with longitudinal cognitive decline and incident dementia in older adults, which might result from disruption of corpus callosum and corona radiata. These findings highlight the value of cholinergic WMHV as a potential indicator of cognitive deterioration.

**Supplementary Information:**

The online version contains supplementary material available at 10.1186/s12877-025-06447-x.

## Introduction

White matter hyperintensities (WMHs) are frequently observed in older adults and their severity tends to increase with age [1, 2]. Research conducted through cross-sectional and longitudinal studies has revealed that WMHs are closely related to cognitive dysfunction [3–6]. Previous studies have identified the WMH volume (WMHV) as a potential biomarker for reflecting cognition. These studies have demonstrated that greater WMHV is associated with worse global and domain-specific cognitive performance, as well as accelerated cognitive decline [3–5]. However, as inter-individual variations and a clinicoradiological discrepancy exist, not all individuals with severe WMH burden experience worse cognitive performance [2, 7]. Furthermore, the location of WMHs appears to be a critical factor in the relationship between WMHs and cognitive function [8, 9]. Evidence implies that WMHs located in strategic white matter tracts, such as the anterior thalamic radiation or forceps minor, are more strongly associated with cognitive performance, compared to WMHs in the whole brain [9–12]. These findings underscore the importance of both WMH volume and location in the development of cognitive dysfunction [2].

The cholinergic system, along with other neurotransmitter systems, plays a crucial role in cognitive function [13–16]. The Cholinergic Pathways HyperIntensities Scale (CHIPS) has been widely used to investigate the association between WMH burden in the cholinergic pathways and cognition [17–20]. Previous studies have shown that executive dysfunction is linked to burden of WMH within the cholinergic pathways rated by CHIPS in patients with Parkinson’s disease or after stroke [19, 20]. Whereas, a study reported that vigilant attention was not related to CHIPS severity in patients after minor stroke [18]. Since CHIPS is a visual rating scale, it is a manual and semi-quantitative method to estimate WMH severity in the cholinergic pathways [17]. The quantitative assessment of WMHs combined with a brain atlas has evolved as a useful and accurate approach to detect and quantify WMHs in the global and regional brain. Few studies take both the volume and location into account in quantitative analysis and explore the association between WMH burden in the cholinergic pathways and cognitive impairment. To address this gap, we hypothesize that WMHV in the cholinergic pathways may be a more practical and novel neuroimaging biomarker compared to traditional whole-brain WMHV, as it could better reflect cognitive function and predict cognitive decline.

In this study, our objective was to investigate the potential Association between cholinergic WMHV and general cognitive function, cognitive decline, and incident dementia over a 5-year follow-up period. We utilized data from a prospective, population-based cohort and estimated WMHV quantitatively across both the entire brain and the cholinergic pathways.

## Methods

### Study participants

This study is a part of the Taizhou Imaging Study (TIS), an ongoing population-based cohort study that aims to investigate risk factors, neuroimaging features, and clinical consequences of age-related diseases. The detailed study protocol has been published previously [21]. Inclusion criteria of TIS were: (1) age between 55 and 65 years; (2) residing in Taizhou for at least the past 10 years; (3) able to walk, communicate, and provide the information independently during the investigation. Exclusion criteria of TIS included: (1) previously physician-diagnosed dementia, stroke, Parkinson’s disease, cancer, psychiatric disorders, or other severe neurological diseases; (2) magnetic resonance imaging (MRI) contraindications or known claustrophobia. Overall, a total of 904 individuals were included at baseline of TIS. For the present study, 153 participants were additionally excluded: 138 individuals with poor performance of the Mini-Mental State Examination (MMSE) at baseline (i.e., ≤ 17 for illiterate individuals, ≤ 20 for participants with 1–6 years of education, and ≤ 24 for individuals with > 6 years of education) [22], and 15 individuals with unqualified WMH segmentation processed images, leaving 751 non-demented participants as the analytic sample.

### MRI acquisition and imaging analysis

MRI scans of all participants were acquired on a single 3.0T scanner (Magnetom Verio Tim scanner; Siemens, Erlangen, Germany). The protocol included a localizer scan; a 3D T1 magnetization-prepared rapid gradient-echo (MP-RAGE) sequence: repetition time/echo time 2300/2.98 ms, flip angle 9 degrees, 176 continuous slices, field of view 256 mm, voxel size 1.0 × 1.0 × 1.0 mm; a fluid-attenuated inversion recovery (FLAIR) sequence: repetition time/echo time 8000/96 ms, flip angle 150 degrees, 70 continuous slices, field of view 220 mm, voxel size 0.9 × 0.9 × 3.0 mm.

Global WMHs were segmented automatedly by a lesion growth algorithm implemented in the Lesion Segmentation Toolbox (LST) version 3.0.0 (www.statistical-modelling.de/lst.html) for SPM12 [23] (Fig. S1A). This algorithm can obtain a WMH binary mask from a combination of T1 and FLAIR images. All WMH segmentation maps were visually inspected. For the segmentation of cholinergic WMHs, firstly, we extracted brain tissue from T1 images using the tool *bet* from FMRIB Software Library (FSL) version 6.0.5 (http://www.fmrib.ox.ac.uk/fsl) [24], and then registered it to a Montreal Neurologic Institute (MNI) 152 T1 template using FSL’s registration tool *fsl_reg*. Next, we normalized the global WMH binary mask in the individual T1 space extracted by LST to the MNI space using the non-linear registration parameters for spatial normalization, to account for inter-individual variability in head size (Fig. S1B). Subsequently, based on previous studies [25], we obtained the cholinergic pathway mask consisting of genu of corpus callosum (GCC), splenium of corpus callosum (SCC), anterior corona radiata (ACR), external capsule (EXCAP), posterior thalamic radiation (PTR), superior longitudinal fasciculus (SLF), and sagittal striatum (SS), using the Johns Hopkins University diffusion tensor imaging-based white matter tract (JHU) atlas as reference [26] (Fig. S2A). Each tract mask in the MNI space was overlaid on the global WMH binary mask in the MNI space to label WMH voxels within tracts (Fig. S1C). The cholinergic WMHV was defined as the voxel size multiplied by the total number of labeled WMH voxels in the cholinergic pathway mask (Fig. S1D). Further, tract-specific WMHV was derived by extracting the labeled WMH voxels in each tract and summing their voxel size. All the cholinergic and tract-specific WMHV were calculated by using FSL’s tool *fslmaths* and *fslstats*.

### Cognitive assessment and diagnosis of dementia

Global cognitive function at baseline was assessed with MMSE. During follow-up, a concise neuropsychological test battery was used to assess global cognition and cognitive domains of memory, attention, executive function, language, and visuospatial function. The test battery included (1) MMSE; (2) Montreal Cognitive Assessment; (3) Auditory Verbal Learning Test; (4) Modified Fuld Object Memory Evaluation; (5) Conflicting Instructions Task and Go/No Go Task; (6) Trail-Making Test A & B; (7) Animal Fluency Test; and (8) Clock Drawing Test. The neuropsychological test battery has been previously detailed and validated in Chinese population-based studies [21, 27].

To estimate cognitive decline, the annual change of cognitive performance was calculated by substracting the baseline score from the follow-up score and then dividing the result by follow-up years. MMSE was subdivided into six cognitive domain scores, i.e., orientation, registration, attention and calculation, recall, language, and constructional ability [28].

Neurologists and neuropsychologists reviewed medical, neurological, and cognitive assessment data, and reached a consensus diagnosis of dementia using the Diagnostic and Statistical Manual of Mental Disorders, Fifth Edition [29].

### Covariates

At baseline, demographical profiles (sex, birth date, and years of education) and vascular risk factors (current smokers, current alcohol drinkers, hypertension, diabetes, and hyperlipidemia) were obtained using structured Questionnaires, physical examination, and laboratory results. Current smokers were defined as regular smoking for at least 6 months before the survey, and current alcohol drinkers were defined as consuming more than three drinks per week for at least 6 months before the survey. Hypertension was defined as mean blood pressure ≥ 140/90 mmHg, or a previous diagnosis of hypertension, or use of anti-hypertensive medications. Diabetes was defined as a fasting plasma glucose level ≥ 7.0mmol/L, or a previous diagnosis of diabetes, or use of anti-diabetic medications. Hyperlipidemia was defined as a serum level of total cholesterol ≥ 5.2 mmol/L, or triglycerides ≥ 1.7 mmol/L, or a previous diagnosis of hyperlipidemia, or use of lipid-lowering medications. Lacunes and cerebral microbleeds were assessed qualitatively according to Standards for Reporting Vascular Changes on Neuroimaging (STRIVE) criteria [30].

### Statistical analyses

Statistical analyses were performed using the R software version 4.2.1 (R Core Team, www.R-project.org). Two-tailed *P* values < 0.05 were considered statistically significant. Continuous variables were presented as mean ± standard deviation (SD), or median with interquartile range (IQR) as appropriate. Categorical variables were summarized as frequencies and proportions. The normality of continuous variables was evaluated using Shapiro-Wilk test. Analysis of variance (ANOVA) or Kruskal-Wallis test was used to compare continuous variables. Pearson’s chi-square test was used to compare categorical variables.

Firstly, multivariable linear regression was used to examine the associations of normalized global and cholinergic WMHV with Z score of MMSE (ZMMSE) and annual change of MMSE and cognitive domain scores. Adjustments were made in three successive models: Model 1 was adjusted for baseline MMSE (only for cognitive change), age at baseline, sex, and years of education. Model 2 was additionally adjusted for lacunes (yes/no), and cerebral microbleeds (yes/no) based on Model 1, since these markers of cerebral small vessel disease (CSVD) co-exist with WMHs in older adults and affect cognitive function [31, 32]. Model 3 was further adjusted for vascular risk factors, including current smokers (yes/no), and current drinkers (yes/no), hypertension (yes/no), diabetes (yes/no), and hyperlipidemia (yes/no). Additionally, considering potential ceiling effects when regressing WMHV on MMSE scores, the Tobit regression model was performed to analyze the association between normalized global and cholinergic WMHV and MMSE scores via the R package “AER” [33]. Secondly, multivariate Cox proportional hazards regression was used to assess the association between normalized global and cholinergic WMHV and incident dementia, using the R package “survival”. Covariates in the adjusted model included baseline MMSE, age at baseline, sex, years of education, lacunes, cerebral microbleeds, and vascular risk factors. Thirdly, we aimed to identify the strategic white matter tract of the cholinergic pathways in which WMHV was associated with baseline global cognitive function, cognitive decline, and incident dementia. Partial correlation tests were performed to evaluate the relation between tract-specific WMHV and different cognitive outcomes, using the R package “ppcor”. Correction for multiple comparisons was performed using false discovery rate (FDR) correction for seven tracts, and a FDR-corrected *P* value < 0.05 was considered statistically significant.

## Results

### Baseline characteristics of the study population

**Fig. 1:**
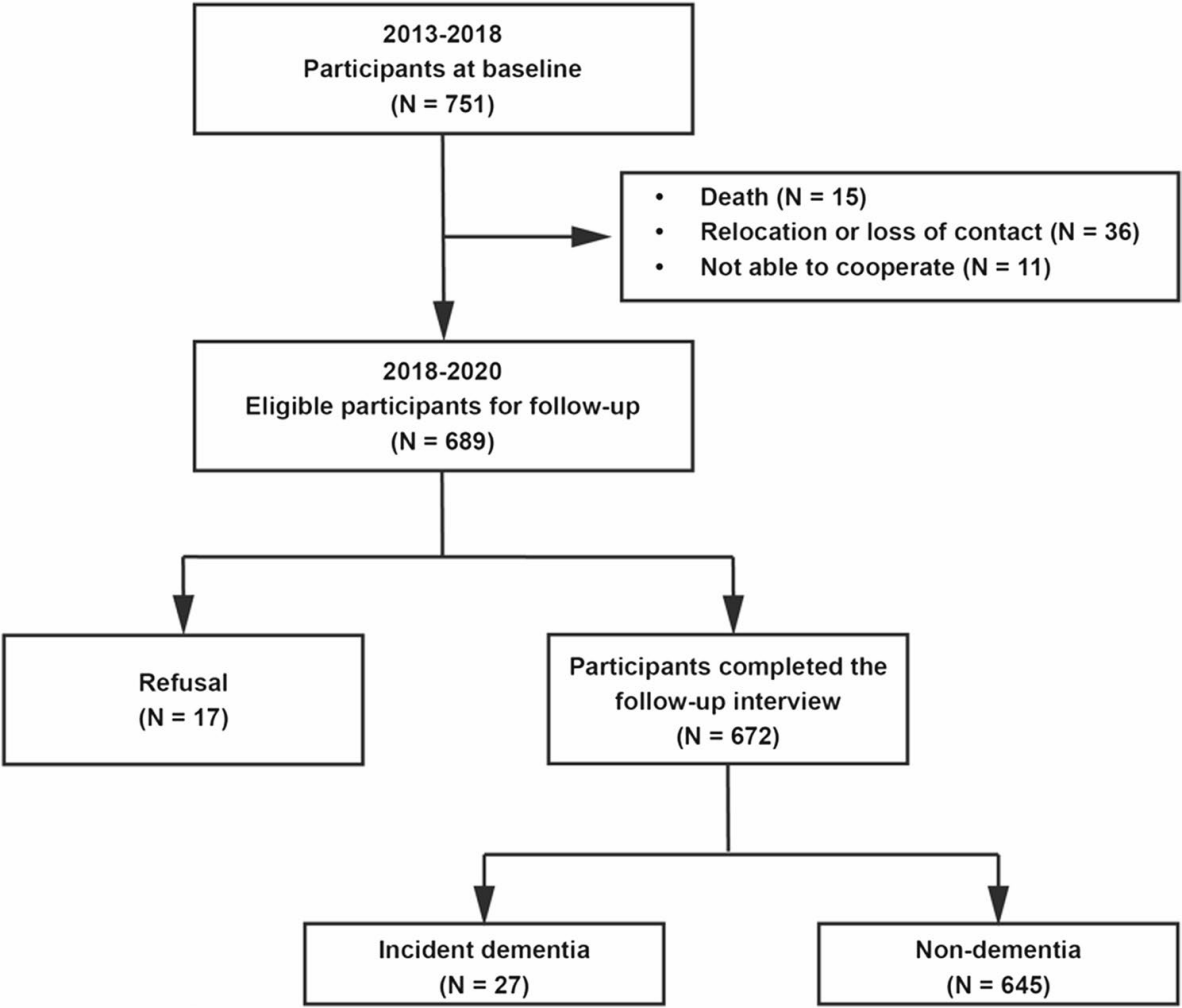
Flow chart of the enrollment and follow-up for the study sample

Table 1 shows the baseline characteristics of the study population, which consisted of 751 participants with a mean age (SD) of 60.07 (2.93) years; 375 of the 751 participants (49.93%) were females. The participants had a median 6 (IQR: 2–9) years of education. The median score of baseline MMSE was 27 (IQR: 25–29). The median of global WMHV was 1.21 (IQR: 0.70–2.18) ml. The median of normalized global WMHV and cholinergic WMHV were 1.62 (IQR: 0.95–2.81) and 0.18 (IQR: 0.09–0.39) ml. As displayed in Supplementary Fig. 2B, WMHs within the cholinergic pathways were prone to distribute in periventricular regions. Among 751 participants at baseline, 672 individuals participated in the follow-up interviews (Fig. 1). We compared baseline characteristics between individuals who were followed up and not followed up, and found no statistically significant differences among the two groups (Table 1).

**Table 1:** Baseline characteristics of study participants

	Total	Followed	Not followed	*p* Value
	(*N* = 751)	(*N* = 672)	(*N* = 79)	
Demographic Characteristics				
Female	375 (49.93)	339 (50.45)	36 (45.57)	0.483
Age, years	60.07 (2.93)	60.09 (2.91)	59.94 (3.19)	0.662
Education, years	6.00 (2.00, 9.00)	6.00 (2.00, 9.00)	6.00 (4.00, 9.00)	0.119
Vascular risk factors				
Current smokers^a^	116 (15.53)	102 (15.25)	14 (17.95)	0.647
Current alcohol drinkers^a^	236 (31.68)	211 (31.49)	25 (33.33)	0.846
Hypertension	371 (49.40)	330 (49.11)	41 (51.90)	0.726
Diabetes	77 (10.25)	66 (9.82)	11 (13.92)	0.347
Hyperlipidemia	399 (53.13)	361 (53.72)	38 (48.10)	0.408
Cognition				
Baseline MMSE, score	27.00 (25.00, 29.00)	27.00 (25.00, 29.00)	28.00 (25.00, 29.00)	0.172
Follow-up MMSE, score	-	26.00 (22.00, 28.00)	-	-
Neuroimaging				
WMHV in the native space, ml	1.21 (0.70, 2.18)	1.23 (0.71, 2.20)	1.15 (0.62, 2.04)	0.309
Global WMHV, ml^b^	1.62 (0.95, 2.81)	1.62 (0.96, 2.82)	1.52 (0.86, 2.65)	0.460
Cholinergic WMHV, ml^b^	0.18 (0.09, 0.39)	0.18 (0.09, 0.40)	0.16 (0.06, 0.30)	0.114
Lacunes	125 (16.62)	111 (16.52)	14 (17.72)	0.911
Microbleeds^a^	113 (15.09)	97 (14.50)	16 (20.25)	0.236
Follow-up year, years	-	5.67 (2.88, 6.84)	-	-

*MMSE* Mini-Mental State Examination, *WMHV* White matter hyperintensity volume

Data represents number (%), mean (standard deviation), or median [interqu artile range]

^a^Participants with missing data: 4 for current smokers; 6 for current alcohol drinkers; and 3 for microbleeds

^b^Global WMHV and cholinergic WMHV are volumes after registration to the MNI space (normalized volumes]

### Association between global and cholinergic WMHV and cognitive performance

To investigate the impact of global and cholinergic WMHV on cognitive impairment, we first examined the association between these two quantitative imaging indices and global cognitive scores. At baseline, neither global nor cholinergic WMHV was significantly cross-sectionally associated with global cognitive function (Table 2 and Table S1). We then analyzed the relationship between global or cholinergic WMHV and longitudinal cognitive change. Over a median 5.67 (IQR: 2.88–6.84) years of follow-up, larger cholinergic WMHV was found to be associated with a greater annual decline of MMSE (β coefficient, −0.239; *P* = 0.004), after adjusting for baseline MMSE, age at basline, sex, years of education, lacunes, microbleeds, and vascular risk factors (model 3). Similarly, the association was also significant between global WMHV and MMSE decline (β coefficient, −0.049; *P* = 0.002) (Table 2).

We further explored the association between global and cholinergic WMHV and decline of cognitive domains. Scores of cognitive domains at baseline and follow-up were listed in Table S2. Decline of cognitive domains was shown in attention and calculation, recall, and language among the study population. After adjusting for baseline MMSE, age at basline, sex, years of education, lacunes, microbleeds, and vascular risk factors, larger cholinergic WMHV was observed to be associated with greater annual change of registration (β coefficient, −0.055; FDR-corrected *P* = 0.048) and constructional ability (β coefficient, −0.037; FDR-corrected *P* = 0.048), but not related to annual change of orientation, attention and calculation, recall, and language. As for global WMHV, there were no significant associations between decline of cognitive domains and this index (Table S3).

**Table 2:** Association between global and cholinergic WMHV and global cognitive function

	ZMMSE		Annual change of MMSE	
	β (SE)	*p* Value	β (SE)	*p* Value
Global WMHV				
Model 1	0.005(0.010)	0.597	−0.052(0.014)	< 0.001**
Model 2	0.006(0.011)	0.564	−0.05(0.016)	0.001**
Model 3	0.008(0.011)	0.462	−0.049(0.016)	0.002**
Cholinergic WMHV				
Model 1	0.024(0.051)	0.641	−0.252(0.072)	0.001**
Model 2	0.03(0.058)	0.601	−0.241(0.083)	0.004**
Model 3	0.038(0.059)	0.521	−0.239(0.084)	0.004**

The β coefficients correspond with the change in cognitive function (i.e., MMSE in Z score and annual change of MMSE) associated with 1 ml change in the independent variable (i.e., global and cholinergic WMHV)

*MMSE* Mini-Mental State Examination, *SE* Standard error, *WMHV* White matter hyperintensity volume

Model 1 is adjusted for baseline MMSE (only for annual change of MMSE), age at basline, sex, and education years, Model 2 is additionally adjusted for lacunes and microbleeds, based on Model 1 Model 3 is further adjusted for vascular risk factors, including current smokers, current alcohol drinkers, hypertension, diabetes, and hyperlipidemia

*P < 0.05, **P < 0.01

### Cholinergic WMHV has a higher risk for incident dementia compared to global WMHV

In this study, 672 participants were followed for a median of 5.67 years, and 27 incident dementia cases were identified. (Table 3). After adjustment for baseline MMSE, demographic variables, imaging markers, and vascular risk factors, individuals with greater global WMHV had a slightly higher incidence of dementia (HR = 1.23; 95%CI: 1.11–1.35) (Table 3). Furthermore, higher cholinergic WMHV also increased the risk of dementia, with a higher hazard ratio (HR = 3.54; 95%CI: 2.05–6.10) compared to global WMHV.

**Table 3:** Association between global and cholinergic WMHV and incident dementia

	Non-dementia (*N* = 645)	Incident dementia (*N* = 27)
Global WMHV		
Model 1	1.00 (reference)	1.18(1.09–1.28)**
Model 2	1.00 (reference)	1.23(1.12–1.35)**
Model 3	1.00 (reference)	1.23(1.11–1.35)**
Cholinergic WMHV		
Model 1	1.00 (reference)	2.36(1.57–3.54)**
Model 2	1.00 (reference)	3.22(1.86–5.57)**
Model 3	1.00 (reference)	3.54(2.05–6.10)**

The coefficients correspond with the risk of incident dementia associated with 1 ml change in the independent variable (i.e., global and cholinergic WMHV)

*WMHV* White matter hyperintensity volume

Model 1 is adjusted for baseline MMSE, age at baseline, sex, and education years, Model 2 is additionally adjusted for lacunes and microbleeds, based on Model 1, Model 3 is further adjusted for vascular risk factors, including current smokers, current alcohol drinkers, hypertension, diabetes, and hyperlipidemia

*P < 0.05, **P < 0.01

### Relationship between tract-specific WMHV within the cholinergic pathways and cognition

**Fig. 2:**
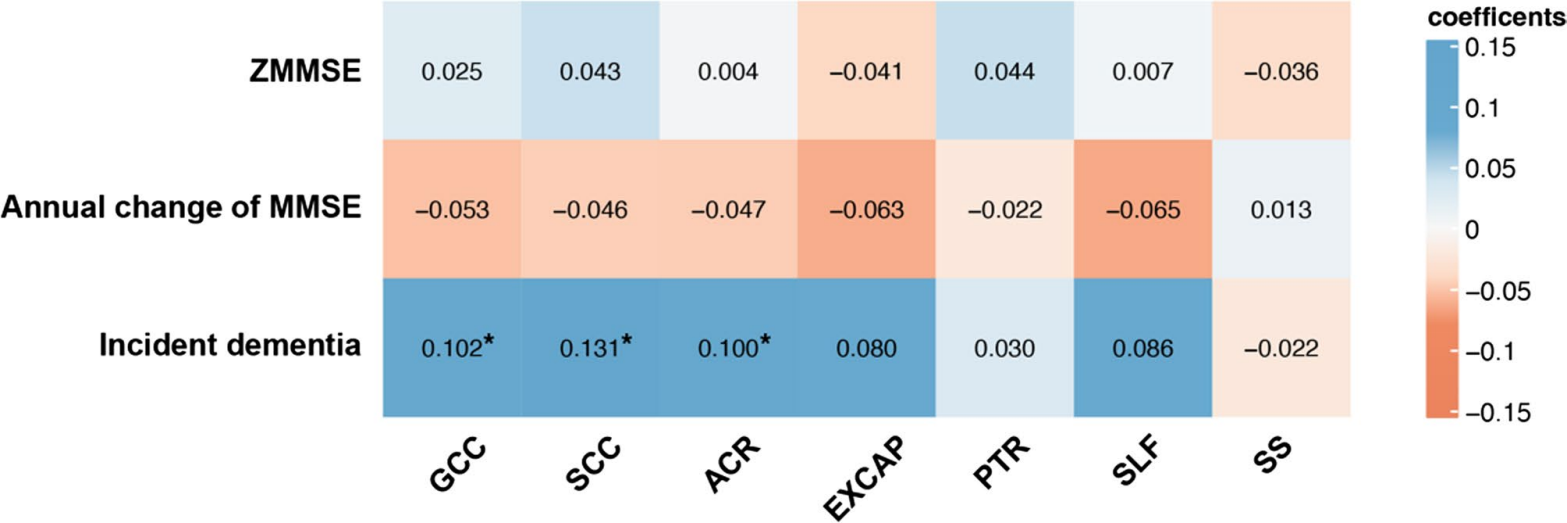
A heat map summarizing partial correlations between tract-specific WMHV within the cholinergic pathways and cognitive outcomes. Baseline MMSE (only for annual change of MMSE and incident dementia), age at basline, sex, education years, lacunes, microbleeds, vascular risk factors, and follow-up years (only for incident dementia) are controlled. *P < 0.05 was considered statistically significant after correction for multiple comparisons using false discovery rate correction. *MMSE* Mini-Mental State Examination, *GCC* genu of corpus callosum, *SCC* splenium of corpus callosum, *ACR* anterior corona radiata, *EXCAP* external capsule, *PTR* posterior thalamic radiation, *SLF* superior longitudinal fasciculus, *SS* sagittal striatum

We further identified the strategic white matter tract of the cholinergic pathways in which WMHV was associated with different cognitive outcomes. As shown in Fig. 2, there were no significant correlations between tract-specific WMHV and ZMMSE. Although we observed a greater annual decline of MMSE in individuals with larger WMHV within six tracts, the correlation was not statistically significant. Interestingly, larger WMHV on GCC, SCC, and ACR within the cholinergic pathways was found to be correlated with incident dementia, after correction for multiple testing, controlled for baseline MMSE, follow-up years, lacunes, microbleeds, demographic and vascular risk factors.

## Discussion

The aim of this study was to investigate the association between cholinergic WMHV and cognitive dysfunction in older adults. Our finding revealed that larger cholinergic WMHV was associated with greater cognitive decline and incident dementia. Additionally, GCC, SCC, and ACR these tracts of the cholinergic pathways were identified as strategic locations correlated with incident dementia. This study indicates that cholinergic WMHV might be a promising neuroimaging biomarker for reflecting cognitive degeneration in a community-dwelling population.

Our results indicate that cholinergic WMH burden was related to global cognitive decline, consistent with previous studies using the CHIPS visual rating score [34–36]. A community-based study using semi-quantitative assessment also found a correlation between cholinergic WMH burden and decline in processing speed and semantic memory over time [25]. In addition, some studies have reported an association between cholinergic WMH burden and memory impairment and decline [25, 37], our study also found that cholinergic WMHV was related to decline in registration and constructional ability. Though global WMHV was found related to global cognitive decline, no associations were found between global WMHV and decline of cognitive domains. These findings imply that cholinergic WMHV might be an early and sensitive indicator of cognitive decline in middle-aged and older adults. However, unlike the findings reported in other cohort studies, we did not find the cross-sectional association between global WMHV and global cognitive function. In community-based cohort studies involving older residents (e.g., the Rotterdam Study, and the Northern Manhattan Study), higher global WMHV was associated with worse cognitive performance in participants aged 65 years or older [3, 38]. This inconsistency could be due to the relatively young age (55–65 years old) at baseline of our study sample. WMHV in the whole brain in this study was relatively low compared to other studies from a community-dwelling population (aged over 65 years) [39–41], as WMH burden typically increases with age [1]. As a result, the associations between global WMHV and cognition may have been underestimated. Furthermore, as reported in previous literature [2, 5], we found that global WMHV was associated with incident dementia. Interestingly, cholinergic WMHV was also found to contribute to incident dementia in this study, which had nearly 3-fold higher risk of incident dementia compared to global WMHV. It is probable that WMHs within the cholinergic pathways may contribute to reduced integrity of cholinergic white matter projection [42], which may make a stronger contribution to cognitive function than nucleus basalis of Meynert volume [16, 43, 44].

The strategic locations within the cholinergic pathways are fundamental to cognitive function. In this study, genu and splenium of corpus callosum and ACR have been found to be correlated with incident dementia. This is in line with previous studies that highlight the important role of corpus callosum and corona radiata in cognitive function. For example, a study on a sample with CSVD showed that lesions on corpus callosum increased the risk of dementia [45]. Several studies based on diffusion tensor imaging (DTI) found that integrity of corpus callosum and ACR could predict cognitive impairment [46–48]. However, we did not find WMHs in these seven tracts of the cholinergic pathways related to global cognitive function and decline. This finding is in keeping with a community-based study that reported WMHV within white matter tracts was not related to global cognition [49]. This might be a result of diverse associations of different fiber tracts with specific-domain cognitive performance [49, 50]. Future studies need to assess cognitive domains and shed light on their relationships with WMHs in different regional tracts within the cholinergic pathways.

It should be noted that this study is one of the few studies to use a quantitative assessment measuring WMHV within the cholinergic pathways. Most of previous studies have relied on the CHIPS visual scale to assess the severity of WMHs in cholinergic pathways [17–20, 51]. Quantitative assessments of cholinergic WMHV vary among studies. In the Sunnybrook Dementia Study, subcortical hyperintensities were accessed with a mask of the lateral cholinergic pathways obtained by a semiautomatic extraction method, whereas the medial cholinergic pathways were not included in the mask [37]. The Northern Manhattan Study measured cholinergic WMH burden semi-quantitatively based on JHU atlas [25]. In another study, Babiloni et al. manually traced the cholinergic pathways in amnesic mild cognitive impairment patients to evaluate volumes of WMHs within the pathways [52]. Our study provides a practical and time-saving measurement only based on T1, FLAIR, and JHU atlas to obtain cholinergic WMHV. However, high-quality DTI data are needed to track the cholinergic pathways and compare them with the pathways obtained from JHU atlas in future studies. Building on the methodology and findings of our study, cholinergic WMHV has the potential to be applied in clinical practice, such as serving as an early warning indicator for high-risk populations with cognitive impairment or as a tool for clinical diagnosis and differential diagnosis of dementia, which warrants further exploration in future studies.

Overall, our study highlights the potential value of cholinergic WMHV as a neuroimaging biomarker for indicating cognitive decline in older adults. The strengths of this study include a large sample size, a relatively long follow-up period, and a Quantitative assessment of WMHV. However, several Limitations also should be noted. First, our baseline cognitive interview only used MMSE to assess global cognitive function. As a result, the ability of cholinergic WMHV to indicate different cognitive domains and their decline could not be fully explored. Further studies should use a comprehensive neuropsychological test battery to estimate cognitive domains and further explore relationships between cognitive domains and cholinergic WMHV. Second, the longitudinal MRI was not performed in this study, leading to the lack of evaluation of WMH progression in the whole brain and the cholinergic pathways. In addition, participants only have one follow-up visit for cognitive assessments in our study, which Limits the analysis of the relationship between imaging biomarkers and the cognitive trajectory. Associations between WMH progression and the cognitive trajectory are a worthy research direction in the future. Third, the participants included in this study were aged 55–65 years old, which may underestimate the Associations between WMHV and cognitive function. Although a proportion of participants were aged 55–59 years at baseline, the vast majority (91.96%) of participants were aged 60 years or older at follow-up. Importantly, our primary conclusions were based on the longitudinal analysis, which ensured the study’s relevance to geriatric research. The inclusion of a few younger participants allowed us to do longitudinal observations and depict the trajectory of cognitive and brain aging in the long and successive follow-ups. We are currently expanding the TIS with a larger age-range study population to establish more general associations. Fourth, our cohort is representative of middle-aged and older adults in rural China, where the overall education level was relatively low due to socioeconomic factors. This limits the generalizability of our findings. Future studies are needed to validate these conclusions in populations with higher education levels. Fifth, Apolipoprotein E ε4 (*APOE* ε4) is an important genetic risk factor for dementia. However, *APOE* ε4 was not included as a covariate in our study, which may have influenced the observed associations between imaging biomarkers and cognition. Our cohort is currently expanding its genetic data collection, and future studies will incorporate *APOE* ε4 to provide more precise estimations of the relationship between imaging biomarkers and cognition. Finally, the cholinergic pathways in this study were identified by structural imaging via MRI and based on previous studies, lacking evidence from molecular imaging biomarkers (e.g., acetylcholinesterase). Multimodal imaging approaches, such as positron emission tomography or single-photon computed emission tomography imaging, and post-mortem MRI of autopsy brains are needed to further identify the cholinergic pathways and verify the mechanism how degeneration of the cholinergic pathways contributes to cognitive impairment.

In conclusion, cholinergic WMHV was associated with longitudinal cognitive decline and incident dementia, which might result from disruption of corpus callosum and corona radiata. This suggested cholinergic WMHV could serve as a useful indicator of cognitive deterioration and has the potential to be integrated into routine neuroimaging protocols in the future. A longitudinal cohort study using a comprehensive battery of cognitive assessment is needed to confirm our findings.

## Supplementary Information

Supplementary Material 1.

## Data Availability

No datasets were generated or analysed during the current study.

## Abbreviations

WMHs: White matter hyperintensities
WMHV: White matter hyperintensity volume
CHIPS: Cholinergic Pathways HyperIntensities Scale
TIS: the Taizhou Imaging Study
MRI: Magnetic resonance imaging
MMSE: Mini-Mental State Examination
MNI: Montreal Neurologic Institute
GCC: Genu of corpus callosum
SCC: Splenium of corpus callosum
ACR: Anterior corona radiata
EXCAP: External capsule
PTR: Posterior thalamic radiation
SLF: Superior longitudinal fasciculus
SS: Sagittal striatum
ZMMSE: Z score of mini-mental state examination
CSVD: Cerebral small vessel disease
