# Supplementary material for: Associations of cholinergic white matter hyperintensity volume with cognitive decline and incident dementia in older adults: a cohort study
Source: BMC Geriatr. 2025 Oct 10;25:768. doi: 10.1186/s12877-025-06447-x (PMC12512280; doi:10.1186/s12877-025-06447-x)
Supplement: Supplementary file 1 — Supplementary Material 1. [file 12877_2025_6447_MOESM1_ESM.docx]

**Table S1.** Association between global and cholinergic WMHV and MMSE scores analyzed via the Tobit model

|  | **MMSE scores** |  |
| --- | --- | --- |
|  | β (SE) | *p* Value |
| **Global WMHV** |  |  |
| Model 1 | 0.017(0.034) | 0.604 |
| Model 2 | 0.019(0.038) | 0.619 |
| Model 3 | 0.027(0.038) | 0.473 |
| **Cholinergic WMHV** |  |  |
| Model 1 | 0.074(0.174) | 0.670 |
| Model 2 | 0.079(0.197) | 0.688 |
| Model 3 | 0.114(0.199) | 0.565 |

*MMSE* Mini-Mental State Examination, *SE* standard error, *WMHV* white matter hyperintensity volume

Model 1 is adjusted for age at baseline, sex, and education years; Model 2 is additionally adjusted for lacunes and microbleeds, based on Model 1; Model 3 is further adjusted for vascular risk factors, including current smokers, current alcohol drinkers, hypertension, diabetes, and hyperlipidemia.

**Table S2.** Scores of cognitive domains at baseline and follow-up

| Cognitive measures (max) | Baseline | Follow-up |
| --- | --- | --- |
| Global cognition (30) | 27.00 (25.00, 29.00) | 26.00 (22.00, 28.00) |
| Orientation (10) | 9.00 (8.00, 10.00) | 9.00 (8.00, 10.00) |
| Registration (3) | 3.00 (3.00, 3.00) | 3.00 (3.00, 3.00) |
| Attention and calculation (5) | 5.00 (4.00, 5.00) | 4.00 (2.00, 5.00) |
| Recall (3) | 3.00 (2.00, 3.00) | 2.00 (1.00, 3.00) |
| Language (8) | 8.00 (6.00, 8.00) | 7.00 (6.00, 8.00) |
| Constructional ability (1) | 1.00 (0.00, 1.00) | 1.00 (0.00, 1.00) |

Cognitive domains were devided from Mini-Mental State Examination (MMSE) following the reference [1].

**Table S3.** Association between global and cholinergic WMHV and annual change of cognitive domains

|  | **Global WMHV** | |  | **Cholinergic WMHV** | |
| --- | --- | --- | --- | --- | --- |
|  | β (SE) | *P* value (FDR) |  | β (SE) | *P* value (FDR) |
| Orientation | -0.009(0.007) | 0.178 |  | -0.030(0.035) | 0.405 |
| Registration | -0.009(0.004) | 0.093 |  | -0.055(0.023) | 0.048* |
| Attention and calculation | -0.009(0.007) | 0.178 |  | -0.030(0.036) | 0.405 |
| Recall | -0.007(0.005) | 0.178 |  | -0.036(0.026) | 0.251 |
| Language | -0.008(0.005) | 0.178 |  | -0.051(0.029) | 0.146 |
| Constructional ability | -0.006(0.003) | 0.093 |  | -0.037(0.014) | 0.048* |

*SE* standard error, *WMHV* white matter hyperintensity volume, *FDR* false discovery rate

Models adjusted for baseline MMSE, age at basline, sex, education years, lacunes, microbleeds, and vascular risk factors.

**P* < 0.05 was considered statistically significant after correction for multiple comparisons using FDR correction for 6 tested cognitive domains.


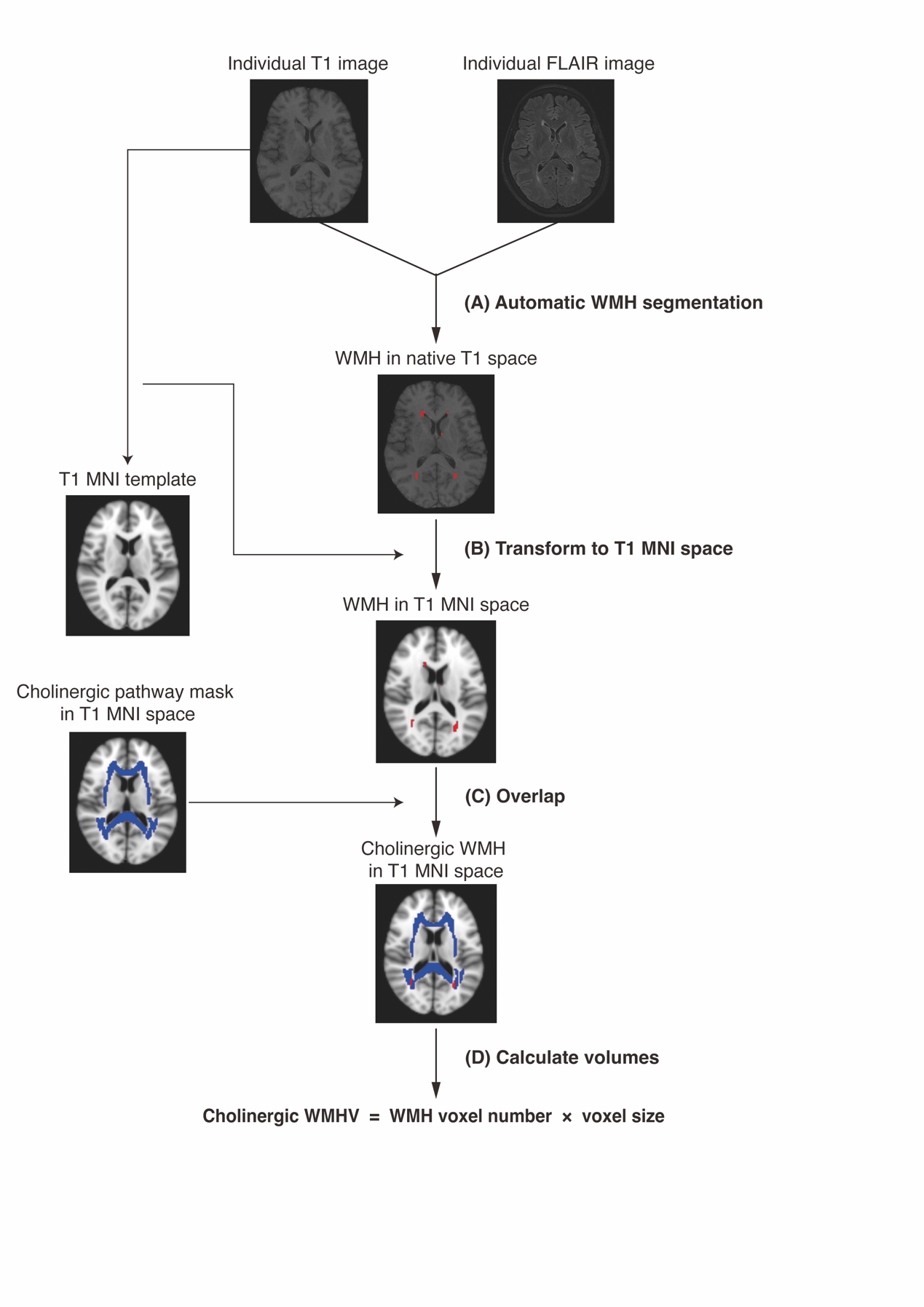


**Fig. S1** Pipeline of cholinergic WMHV estimation. *WMH* white matter hyperintensity, *MNI* Montreal Neurological Institute, *WMHV* white matter hyperintensity volume


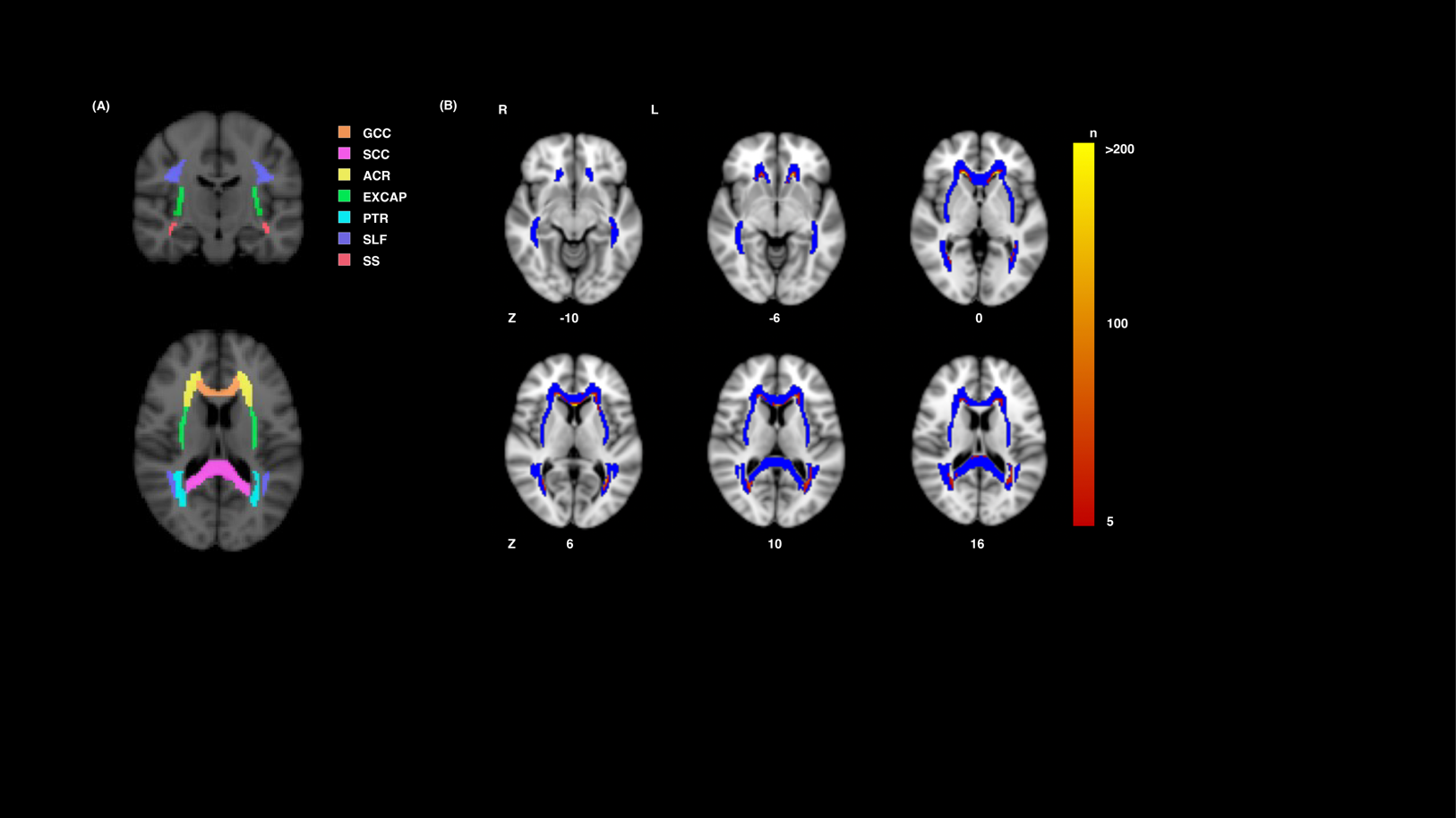


**Fig. S2** The cholinergic tracts and distribution of WMH within the cholinergic pathways. (A) The cholinergic pathways in this study consist of these 7 white matter tracts. (B) Among 751 participants at baseline, voxels with WMH in at least 5 individuals are projected to the cholinergic pathways (blue) on a MNI 152 T1 template. *GCC* genu of corpus callosum, *SCC* splenium of corpus callosum, *ACR* anterior corona radiata, *EXCAP* external capsule, *PTR* posterior thalamic radiation, *SLF* superior longitudinal fasciculus, *SS* sagittal stratum

**References**

1. Cardoso S, Barros R, Maroco J, de Mendonca A, Guerreiro M: Different MMSE domains are associated to cognitive decline and education. *Appl Neuropsychol Adult* 2022:1-7.
